# Performance Evaluation of a Carbon Nanotube Sensor for Fatigue Crack Monitoring of Metal Structures

**DOI:** 10.3390/s20164383

**Published:** 2020-08-06

**Authors:** Shafique Ahmed, Thomas Schumacher, Erik T. Thostenson, Jennifer McConnell

**Affiliations:** 1 Echem Consultants LLC, Poughkeepsie, NY 12601, USA; sahmed@e2chem.com; 2 Civil and Environmental Engineering, Portland State University, Portland, OR 97201, USA; 3 Mechanical Engineering and Materials Science, University of Delaware, Newark, DE 19716, USA; thosten@udel.edu; 4 Civil and Environmental Engineering, University of Delaware, Newark, DE 19716, USA; righman@udel.edu

**Keywords:** carbon nanotube sensor, self-sensing composite, structural health monitoring, metal, fatigue crack, crack propagation, fractography

## Abstract

This article describes research that investigated the ability of a carbon nanotube (CNT) sensor to detect and monitor fatigue crack initiation and propagation in metal structures. The sensor consists of a nonwoven carrier fabric with a thin film of CNT that is bonded to the surface of a structure using an epoxy adhesive. The carrier fabric enables the sensor to be easily applied over large areas with complex geometries. Furthermore, the distributed nature of the sensor improves the probability of detecting crack initiation and enables monitoring of crack propagation over time. Piezoresistivity of the sensor enables strains to be monitored in real time and the sensor, which is designed to fragment as fatigue cracks propagate, directly measures crack growth through permanent changes in resistance. The following laboratory tests were conducted to evaluate the performance of the sensor: (1) continuous crack propagation monitoring, (2) potential false positive evaluation under near-threshold crack propagation conditions, and (3) crack re-initiation detection at a crack-stop hole, which is a commonly used technique to arrest fatigue cracks. Real-time sensor measurements and post-mortem fractography show that a distinguishable resistance change of the sensor occurs due to fatigue crack propagation that can be quantitatively related to crack length. The sensor does not show false positive responses when the crack does not propagate, which is a drawback of many other fatigue sensors. The sensor is also shown to be remarkably sensitive to detecting crack re-initiation.

## 1. Introduction

Metal structures exposed to cyclic loading, including aerospace structures, bridges, and vibrating machinery, are susceptible to fatigue cracking. Monitoring fatigue cracks in metal structures is thus critically important for preventing failure by making repair decisions before a crack reaches its critical length, leading to fracture. The literature shows that material fatigue contributes to the majority of in-service mechanical failure of metal structures [1,2,3]. Furthermore, confronting material fatigue is expensive. In the United States (U.S.), an estimated 3% of the gross national product [4,5,6] is spent on replacing, repairing, inspecting, and/or monitoring fatigue damaged structures. Conventional fatigue repair techniques, including drilling crack-stop holes [7,8], adding bolted steel plates [9,10], and adhesively bonding fiber-reinforced polymer (FRP) sheets [11,12,13], often fail to reliably arrest a fatigue crack, and may result in crack re-initiation, which may trigger catastrophic failure.

### 1.1. State-of-the-Art in Metal Fatigue Crack Sensing

Various sensing approaches have been studied for detecting and monitoring fatigue cracks in structures, including using crack propagation gages (CPGs) [14,15], electrochemical fatigue sensors (EFSs) [16,17], meandering winding magnetometers (MWMs) [17,18,19], large area electronics (LAE) strain sensing sheets [20,21], piezoelectric transducers, ultrasonic sensors [2,3], fiber Bragg grating ultrasonic sensors [22], thin film (styrene-ethylene/butylene-styrene - carbon black) sensors [23], and acoustic emission (AE) monitoring [24,25,26].

Crack monitoring effectiveness of CPGs and EFSs depends on the direct alignment of the sensor with the direction of crack propagation. Additionally, proper bonding of the CPG is crucial to avoid the situation where the crack in the substrate propagates without breaking the resistance strands or the resistance strands break before the crack passes through it, resulting in measurement errors [14]. Flexibility and durability are concerns for the EFS, which uses an electrolyte that eventually dries out. Furthermore, the EFS uses a purely empirical algorithm to detect fatigue crack initiation [27,28], which may only apply for the specific situations in which it has been evaluated. Finally, mounted at an existing crack tip, the electrochemical fatigue crack sensor is small and designed to detect crack initiation; it is not suitable for monitoring crack propagation. Eddy current-based sensors can detect microcracks in metallic structures using proximity sensing [29]. MWMs (using eddy currents) can reliably detect buried and surface cracks. An MWM-array sensor is most effective when the crack is underneath the sensor [30]. The MWM-array sensor system uses a measurement technique that may require expensive hardware and specialized software to acquire and interpret the raw data for crack monitoring.

LAE strain sensing sheets can potentially monitor fatigue crack activity over a large area. The arrangement of each sensor in an LAE sheet can influence the measured strain field. Yao and Glisic [20] showed that a fatigue crack could be tracked visually using a contour map of measured strains complementary to using the response of individual sensors in the LAE sheet. It should be noted that electrical resistance strain gauges have a limited fatigue life depending on the strain level [31]. Similar to traditional electrical resistance strain gauges, LAE strain sensing sheets and CPGs may have a limited fatigue life. Thus, they may provide false positive responses under near-threshold loading conditions. Environmental factors and loading conditions in the field can influence the accuracy of AE and fiber Bragg grating ultrasonic fatigue crack monitoring approaches. A primary challenge of these techniques to detect fatigue cracks is accounting for temperature effects and changing load conditions [2]. While AE monitoring can provide some useful information regarding the crack activity, reliability of predicting crack propagation depends on the data processing technique [25]. Moreover, noise in laboratory and field settings can make it challenging to detect crack activity reliably [25]. Ihn and Chang [32,33] used thin dielectric films, which consist of piezoelectric sensors (working as sensors and actuators), to detect and monitor fatigue in a metal substrate underneath a boron fiber/epoxy patch using ultrasonic guided Lamb waves. The primary hurdle to using ultrasonic techniques to detect fatigue cracks is incorporating the effects of load and temperature changes on the ultrasonic response [2].

To be practical, a sensing system must be low-cost, flexible for application to a wide range of fatigue sensitive details, durable, and reliable over the years of anticipated service life. In addition, some conventional repair techniques cover the crack, e.g., bolted steel plates or adhesively bonded FRP sheets. To monitor a fatigue crack underneath a steel or FRP plate, it would be ideal to integrate a sensor between the substrate and bolted plate or embed it into the bond line, respectively. While some commercially available sensors work under specific conditions and situations (e.g., existing crack, particular geometry, no simultaneous retrofitting), none of them meet all of the desired criteria above.

### 1.2. Carbon Nanotube (CNT)-Based Sensors

Carbon nanotubes (CNTs) have been demonstrated to be effective as piezoresistive sensors that enable high sensitivity to the detection of deformation and damage in structures. Specifically, CNT-based thin films have been shown to work well as strain sensors [34,35,36,37,38]. The small size of the nanotubes combined with their ability to create electrically conductive networks offers the potential to detect deformation and damage of composite materials in situ and in real time. For example, damage in fiber composite materials can be monitored by dispersing carbon nanotubes in them [39,40,41,42]. CNT-based sensors have also been used for monitoring interfacial integrity between substrate and adherents [43,44,45].

### 1.3. Fiber-Based CNT Distributed Sensing Network

The sensor used in this work combines the concept of networks of CNTs as piezoresistive sensors with the use of a nonwoven carrier fabric carrier to easily deploy the sensor in applications that cover large areas and complex geometries. The fabric acts as a carrier for the electrically conductive sensing network, which has been utilized as a sensor in the bondline of metal–composite hybrid structures in the authors′ prior work. This prior work focused on utilizing this sensor to monitor strain [38,45] and debonding in the adhesive layer between a metal substrate and an FRP sheet [45]. Dai, et al. [38] demonstrated the repeatability and sensitivity under tensile loading conditions, and also examined the influence of manufacturing on the response of these sensors. Ahmed, et al. [45,46] showed the measurement consistency of the CNT sensor embedded in the adhesive bondline between a structural composite and a steel substrate. Dai [47] investigated the response of the sensor to varying temperature conditions. The exact thermoresistive response depends on the sensor as well as the substrate to which it is bonded, and the temperature coefficient of resistance (TCR) for these sensors is typically smaller than a traditional resistive strain gauge. Because the sensor is both thin and flexible, it can be readily embedded into the adhesive bondline of a metal–composite structural repair [45,46]. The sensor is also low-cost, as commercially available CNTs are localized on the fabric carrier surface requiring a minimal quantity of CNTs.

**Figure 1:**
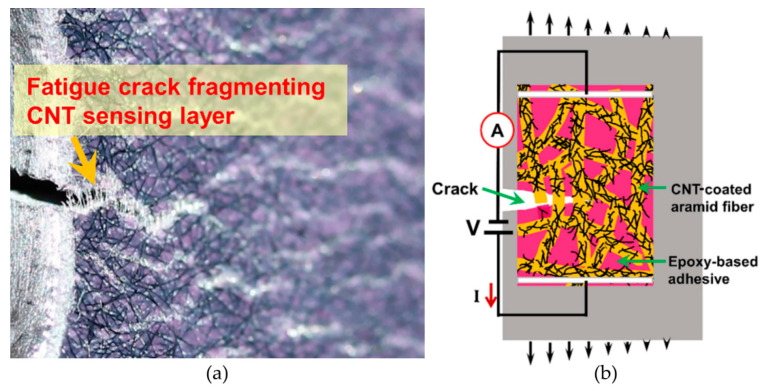
Illustration of carbon nanotube (CNT)-based fatigue sensor concept: (**a**) photo of crack fracturing the sensor and (**b**) sensing principle using electrical resistance measurements across applied electrodes (white lines).

In this article, the concept of fiber-based CNT distributed sensing networks is used as a distributed crack sensor without external structural FRP reinforcement. Figure 1 illustrates the concept—a fatigue crack propagates in the substrate and the sensor fragments with it, thereby severing conductive pathways by stripping the nanotube conductive nanotube film off of the fiber carrier (Figure 1a). The resistance change of the sensor is measured across applied electrodes (white lines in Figure 1b), and the signal resulting from crack propagation is readily distinguishable from resistance change due to strain or deformation [48]. The fracturing of the sensor also allows for visual inspection of fatigue cracks. Moreover, using multiple electrodes located at the boundary of a sensor combined with electrical impedance tomography (EIT) can allow for spatial mapping of damage [49,50].

### 1.4. Motivation and Objectives

In previous work, continuously propagating crack growth scenarios were used to demonstrate the capabilities of various distributed sensors for monitoring fatigue cracks [20,48,51,52]. The present research evaluated the effectiveness and reliability of fatigue crack sensors under various real-life scenarios in the laboratory, including crack propagation, near-threshold crack propagation, and crack re-initiation from a crack-stop hole. The objectives of these tests were to demonstrate that the sensor can detect the arrival of a fatigue crack underneath the sensing layer and quantify crack growth or lack thereof under a variety of realistic loading scenarios.

## 2. Experimental Methods

### 2.1. Materials and Manufacturing

**Figure 2:**
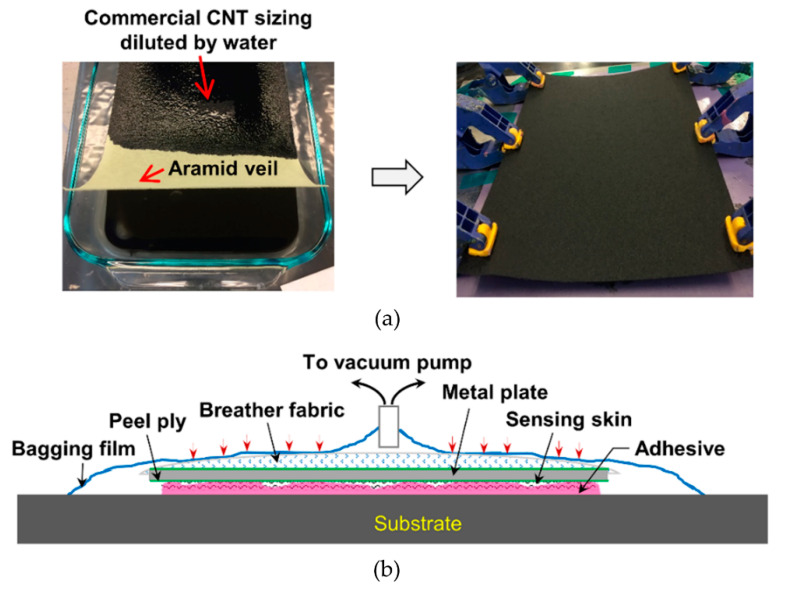
Fabrication process of proposed CNT fatigue sensor: (**a**) dipping aramid veil into CNT sizing agent and drying in the oven; (**b**) implementation of vacuum bagging technique to bond sensing layer to metal substrate.

The CNT sensing layers were fabricated following a procedure described in detail in the authors′ previous work, which involves dip-coating a randomly oriented nonwoven aramid veil (Technical Fiber Products, Schenectady, NY, USA) in a CNT-based sizing agent (SIZICYL XC R2G, Nanocyl, Sambreville, Belgium) followed by drying to create the CNT-based composite coating of nanotubes and other polymers on the fiber surface [35,45,48]. This dip-coating process is illustrated in Figure 2a. After drying the CNT-coated aramid veil, it was trimmed to the desired size sensing layer, 95 × 210 mm. The thickness of the sensor was approximately 300 μm. The edges of the sensing layer, where the electrodes were to be applied, were masked using pressure-sensitive high-temperature masking tape.

ASTM E647 [53] compact tension (CT) specimens were made from an A36 structural steel plate and the surface of the specimen was prepared for adhering the sensing layer by sandblasting, degreasing with alcohol, and applying a thin adhesive layer (HYSOL 9309.3NA, Henkel Corporation, Bay Point, CA, USA) for creating an electrically insulating barrier between the steel substrate and sensing layer. After curing the insulating adhesive layer for 30 min using a 500-watt halogen light, the sensing layer was adhesively bonded using an epoxy adhesive and the vacuum bagging technique, as illustrated in Figure 2b. Subsequently, the sensing layer was cured in a convection oven at 82 °C for one hour, as recommended by the manufacturer. After curing of the adhesive, the masking tape was removed and strip electrodes were applied using silver paint (Flash Dry, SPI Supplies, West Chester, PA, USA) to allow measurement of electrical changes in the sensing layer.

**Figure 3:**
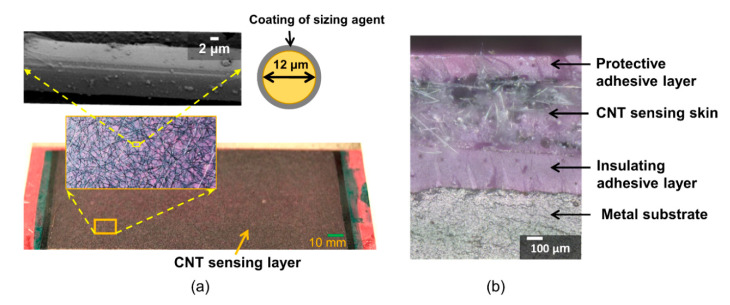
(**a**) Images of CNT fatigue sensor bonded to metal substrate; insets show network of CNT-coated aramid fibers, typical CNT-coated aramid fiber, and schematic of its cross-section; (**b**) photo of interface between sensing layer and metal substrate (before application of silicone paste).

Figure 3a is a photograph showing the CNT sensing layer bonded to the steel specimen and the inset figures show the various levels of the hierarchy of the sensor from the macroscopic sensing layer to the nonwoven fibers to a single fiber coated with a film of CNTs. After bonding the sensing layer, a thin adhesive layer was applied to protect the sensor from the environmental effects. The protective adhesive layer was cured under halogen lights for at least 30 min. Figure 3b shows a cross-section of the sensor where the sensing layer is insulated from the steel substrate with a thin layer of adhesive and protected from the environment with a thin layer on the opposite side. Finally, a layer of silicone paste was applied to prevent any fluid to percolate through the potential pores of the protective adhesive layer, i.e., for weatherproofing in realistic environments. For practical applications where the sensor may be deployed in the field, a procedure could be developed to cure the adhesive at ambient temperatures and eliminate the need for vacuum.

### 2.2. Specimen Configuration

**Figure 4:**
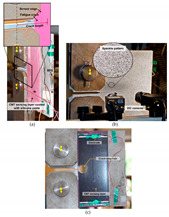
Test setup with ASTM E647 contact tension (CT) specimen instrumented with CNT sensor and back-face strain (BFS) gage: (**a**) fatigue-cracked specimen with inset showing fatigue crack and crack length measurement, (**b**) three-dimensional digital image correlation (3D-DIC) setup for the potential false positive evaluation test specimens, and (**c**) crack-stop hole test specimen to examine fatigue crack re-initiation (shown without protective outer adhesive and silicone layers). Yellow arrows in (**a**), (**b**), and (**c**) show load application points.

The CT specimens (specimen dimensions: W = 203 mm; thickness, B = 12.5 mm shown in Figure 4a) were instrumented with the CNT sensing layer, and a traditional foil strain gage (Model WK-06-250BG-350, Vishay Measurements, VPG, Malvern, PA, USA) was bonded on the back edge of each specimen (gage location is shown in Figure 4a) to measure back-face strain (BFS), which was used to compute fatigue crack length during experimental testing. The strain gage was selected so that it would not reach its fatigue life during the expected strain conditions during the test. The inset in Figure 4a shows a magnified view of the area around the crack, defining crack length as the distance between the line of load application (yellow arrows) and the location of the crack tip. Three-dimensional digital image correlation (3D-DIC) can be used to measure strain field and crack mouth opening displacement (CMOD) [54,55]. A speckle pattern on the opposite side of the specimen, as shown in Figure 4b, was used to measure the strain field and CMOD in the continuous crack propagation monitoring and potential false-positive evaluation tests.

### 2.3. Experimental Procedure and Measurements

Three experimental tests were conducted to evaluate the performance of the CNT-based fatigue sensor: (1) continuous crack propagation monitoring, (2) potential false positive evaluation, and (3) crack re-initiation detection. For all three tests, a sensing layer was bonded to each of the specimens using the technique described in Section 2.1. The sensor was trimmed around the crack-stop hole for the crack re-initiation detection test (Figure 4c). The initial resistance of the sensors bonded on the specimens for experiments, (1) continuous crack propagation monitoring, (2) potential false positive evaluation, and (3) crack re-initiation detection, were 3148, 2731, and 5369 Ω, respectively.

**Figure 5:**
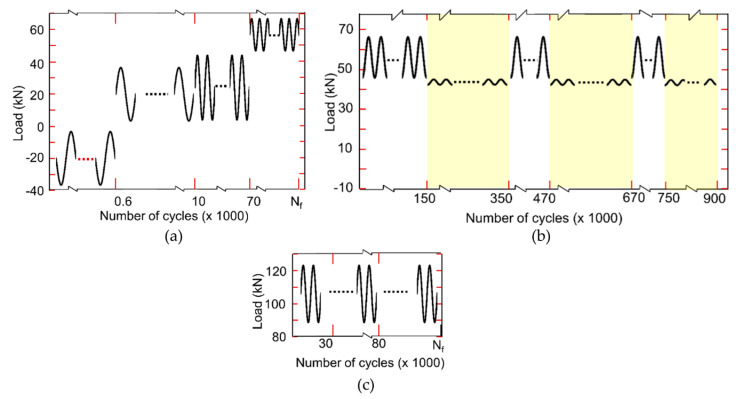
Loading protocols to evaluate the response of the proposed sensor during (**a**) continuous crack propagation monitoring test, (**b**) potential false positive evaluation test, and (**c**) crack re-initiation detection test, specimen with a crack-stop hole.

The loading protocol for the continuous crack propagation monitoring test is illustrated in Figure 5a; it satisfies linear elastic fracture mechanics (LEFM) conditions, which are required to compute crack length using BFS measurements. First, compression–compression constant amplitude (CA) cyclic loading (Cycles 1 to 600) was applied to initiate a crack at the notch tip, followed by tension–tension CA cyclic loading (Cycles 601 to 70,000) with a load ratio, R = P_min_/P_max_ ≈ 0.1 to grow the crack to a length, a = 53 mm. P_min_ and P_max_ represent minimum and maximum peak load amplitudes, respectively. Note that after 10,000 cycles the loading frequency was increased from 1 to 2.5 Hz and Pmax slightly increased to accelerate the test. Subsequently, tension–tension CA cyclic loading with a load ratio, P_max_ = 66.7 kN and R = 0.67, was applied to grow the crack until the specimen fractured.

Figure 5b illustrates the loading protocol for the potential false positive evaluation test, which consisted of two regimes, namely crack growth and near-threshold (segments shaded in yellow). Testing conditions for this test also satisfy LEFM conditions. During the crack growth segments, tension–tension CA cyclic loading with P_max_ = 66.7 kN and R = 0.67 was used. Near-threshold segments were quantified by P_max_ = 45 kN and R = 0.95, which are close to, but below, the anticipated crack propagation threshold, i.e., no crack growth was expected during these loading segments. Throughout the test, a loading frequency of 2.5 Hz was used. Note that this specimen was pre-cracked according to ASTM E647 prior to testing (loading not shown in Figure 5b), i.e., the potential false positive evaluation test started with an existing crack of length, a = 53 mm.

For all tests, the sensing response of the CNT-based fatigue sensor was recorded using a Keithley 6430 voltage–current meter (Keithley Instruments, Inc., Cleveland, OH, USA) by sourcing a constant voltage of 20 V and measuring the current using a two-wire technique employing a sampling rate of 12.5 Hz. Furthermore, BFS measurements were acquired with a System 8000 (Vishay Precision Group, Inc., Malvern, PA, USA ) data acquisition system and StrainSmart^®^ software (Version 8000). For the potential false positive evaluation test, a series of images were collected from the speckle-patterned side of the CT specimen using a 3D-DIC system (Correlated Solutions, Inc., Irmo, SC, USA) of each crack propagation segment (non-shaded areas in Figure 5b). The specimen was reloaded between segments from 0 to 66.7 kN and load was held at intervals of 4.45 kN for taking DIC images.

**Figure 6:**
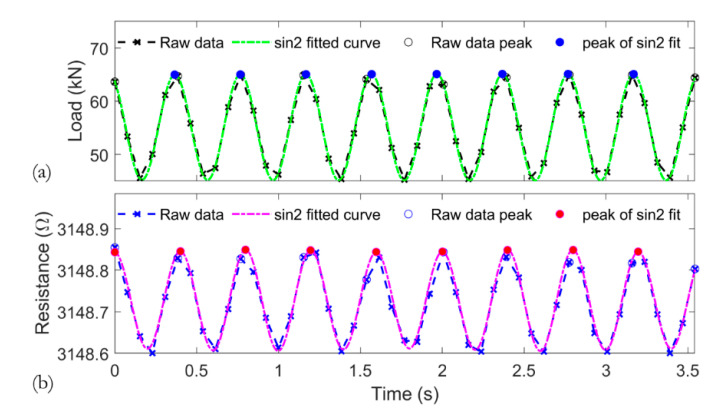
Sample data with selected peaks for both raw and sine curve-fitted data: (**a**) load vs. time and (**b**) resistance vs. time.

Processing of the collected data included selecting peak values from the cyclic response. Figure 6 shows sample load and resistance data. Peak values were determined using the “findpeaks” function in MATLAB. The sampling rate of 12.5 Hz was not fast enough to accurately capture the sinusoidal shape of the applied load (Figure 6a), which had a frequency of 2.5 Hz, and this also manifested in the sensor′s response (Figure 6b). Alternatively, the peak values were selected from a sine curve that was fitted to the raw data capturing 10 consecutive peaks. Due to the curve fit, there might be slight variations in the exact time of the load and sensor response peaks. Therefore, all data are presented in terms of the number of cycles and not time. The differences between selected peak values were found to be minor, and computational time could be saved by selecting peaks from the raw data. Hence, the latter approach was used for further processing.

Although not further discussed in this article, it can be observed that the CNT sensor is capable of sensing each individual load cycle, highlighting the sensor′s piezoelectric sensing capabilities to monitor strain variations.

Finally, cyclic loading was periodically paused to create visible beach marks on the fracture surface. After testing, the crack surface was cut from the specimen for fractographic analysis at various magnifications. Beach mark locations were measured directly from the crack surface using a caliper as well as scaled photographs. High magnification images were obtained using a scanning electron microscope (JEOL USA, Inc., SEM–JSM-7400F, Peabody, MA, USA) with 3 kV accelerating voltage to document fatigue striations. An image processing algorithm was developed to analyze the SEM micrographs for estimating spacing between fatigue striations.

## 3. Results and Discussion

In this section, the results of all three experimental tests are presented and discussed in individual subsections. Results include damage quantifications, forensic investigations, and the responses of the CNT-based sensor. Fatigue crack lengths were calculated for each cycle using the BFS measurements and verified via forensic investigations of fracture surfaces. Relationships between crack length and CNT sensor response are presented and discussed to demonstrate the capability of the proposed CNT-based fatigue sensor.

### 3.1. Continuous Crack Propagation Monitoring Test

**Figure 7:**
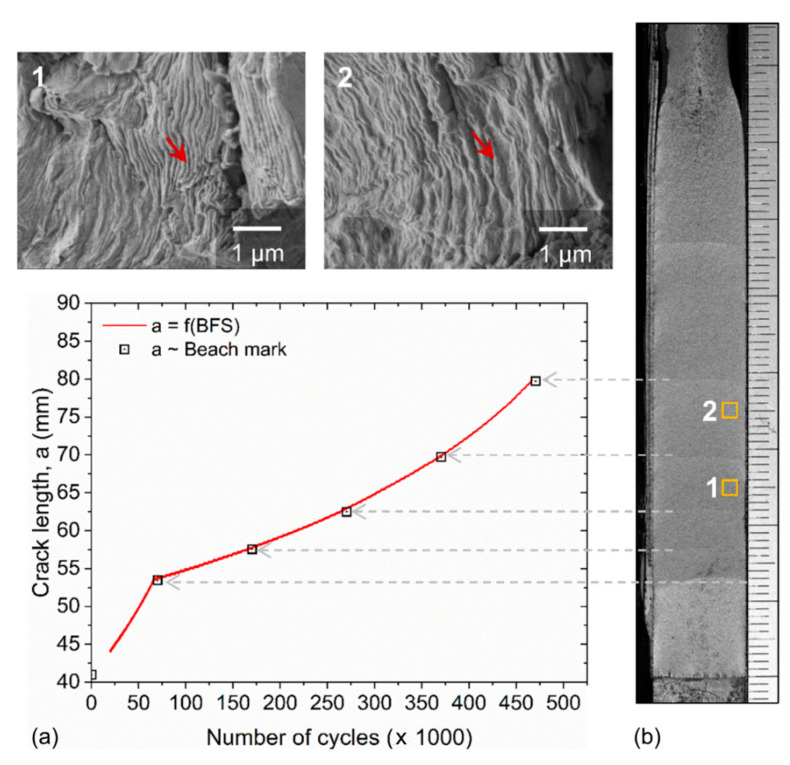
Fractographic approach to validate calculated crack length using BFS data. The figure shows (**a**) a plot of crack length vs. number of load cycles with (**b**) an optical image showing the corresponding beach marks directly observed on the fracture surface. The high-magnification SEM micrographs (insets 1 and 2) show locations 1 and 2 of fatigue striations with different spacings (red arrows in each inset point to an individual striation).

The objective of this test was to determine how accurately the proposed sensor can track the arrival and propagation of a fatigue crack in a metal. BFS measurements were used to predict crack length using the compliance equation proposed by Newman, et al. [56]. The shape and position of successive crack fronts were visible by the beach marks, which were created in the fatigue fracture surface during the loading pauses and are shown in Figure 7. These beach marks were used to validate the accuracy of the calculated crack lengths. The graph in Figure 7 shows excellent agreement (R^2^ = 0.999) between calculated crack length (solid line) and observed beach marks (square marks). To the right of the graph is an optical image of the fracture surface showing the position of the beach marks and their corresponding position on the graph. Positions 1 and 2 in the optical image were examined under high magnification to study fatigue striations (see insets 1 and 2 in Figure 6). Individual striations, which are the finely banded marks, can clearly be observed. These result from the fatigue crack growing a small amount during each load cycle. The micrographs of locations 1 and 2 show significantly different spacings of the individual striations (examples labeled with red arrows), with location 2 showing a much larger spacing, which is a result of higher stress intensity associated with the larger crack length. The average spacing of the striations shown in insets 1 and 2 were found to be 8 × 10^−8^ m/cycle and 15 × 10^−8^ m/cycle, respectively. These measurements correspond reasonably to the crack growth rates calculated from the BFS measurements, which are 7 × 10^−8^ m/cycle and 12 × 10^−8^ m/cycle. These observations also provide evidence of a stable crack growth rate under the applied cyclic loading.

**Figure 8:**
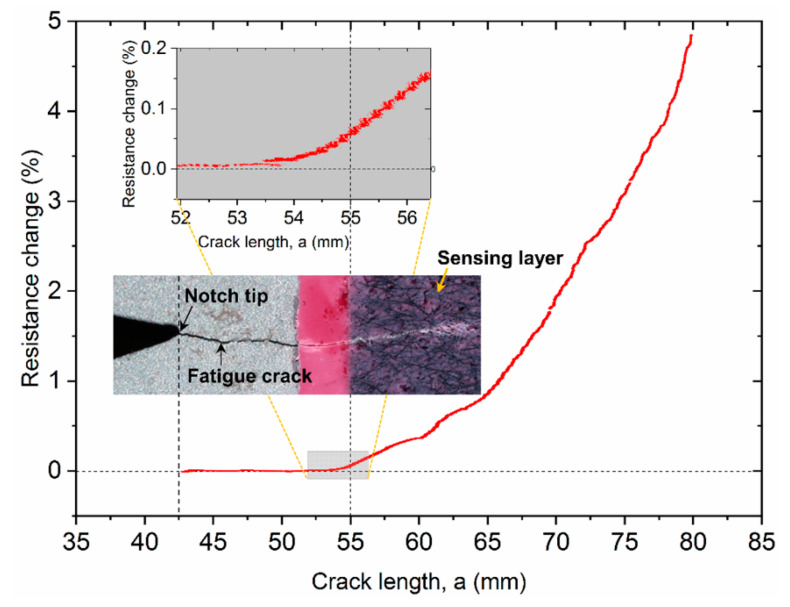
Normalized resistance change vs. crack length. Inset: photograph of the propagating fatigue crack scaled with the *x*-axis and a zoomed-in view of resistance change vs. crack length near the sensor edge (shaded graph).

Figure 8 shows a graph of the measured resistance change of the sensor and crack length. The arrival of the fatigue crack at the sensor can be accurately detected based on the distinguishable resistance change of the sensor. In this test, the fatigue crack reached the edge of the sensor at a crack length, a = 55 mm. Figure 8 (inset) shows a photograph of the crack tip and a close-up view of resistance change near the region of the sensor edge. After the fatigue crack arrives at the sensor, its resistance change gradually increases as the fatigue crack continues to propagate. This observation highlights the sensor′s ability, for this scenario, to monitor fatigue crack arrival and growth. In fact, the sensor detects the presence of the crack slightly before the crack tip reaches the sensor (see shaded inset graph in Figure 8). This early detection is likely due to either the high local stress concentration at the crack tip creating a large plastic zone, which may disrupt the nanotube network locally, or tunneling of the crack in the center of the specimen (note that the slightly curved beach marks in the optical image of Figure 7 support this hypothesis that the crack front does not move uniformly through the thickness of the specimen). The photograph in Figure 8 shows how the sensor also cracks along with the fatigue crack in the substrate, allowing for direct visual inspection of crack growth, which is an advantage compared to other distributed sensors.

### 3.2. Potential False Positive Evaluation Test

**Figure 9:**
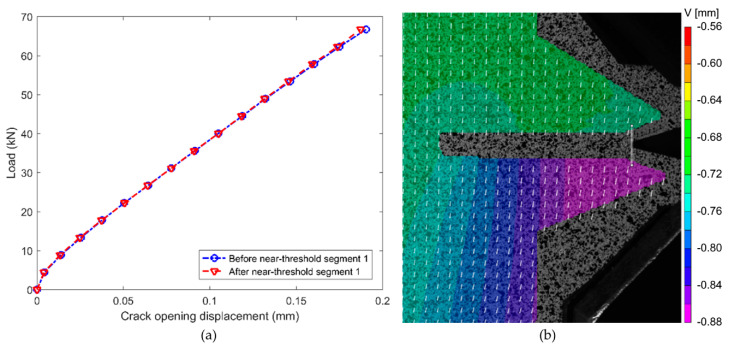
(**a**) Crack opening displacement before and after near-threshold loading cycles. (**b**) 3D-DIC displacement contour plot. White line with circular ends shows where crack mouth opening displacements (CMODs) were calculated and white arrows represent the displacement vector field.

False positive responses of crack propagation are a drawback of many other fatigue sensors (see discussion in Section 1.1). This test aimed to determine the CNT sensor′s reliability under near-threshold crack propagation conditions. In this test, the near-threshold regimes were created by selecting lower magnitude CA sinusoidal cyclic loads (load segments shaded in yellow in Figure 5b) intermittent with higher amplitude loadings that were designed to result in crack propagation. Figure 9 shows a comparison between select CMODs measured using 3D-DIC before and after each selected near-threshold loading cycle segment. It can be observed that these two sets of measurements are virtually identical (the correlation coefficient and the maximum relative difference between these arrays are 1.0 and 0.1%, respectively), demonstrating that the crack remains stationary during the near-threshold load cycles.

**Figure 10:**
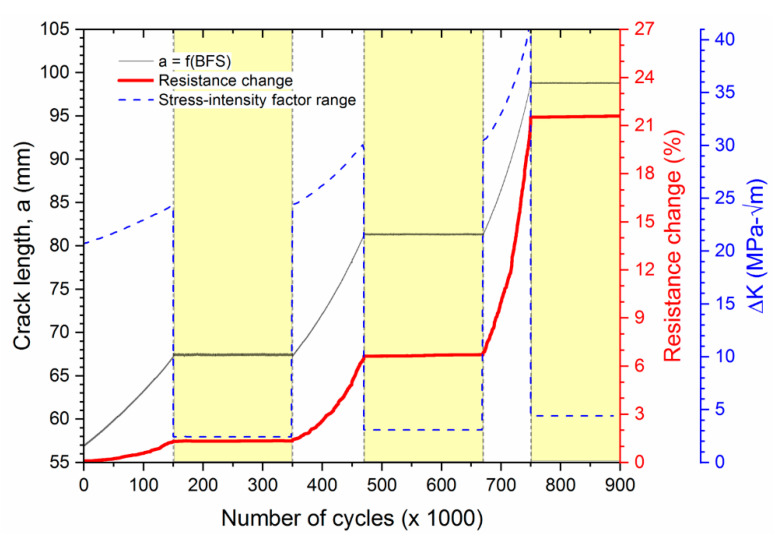
Crack length, electric resistance changes of the CNT sensor, and range of stress intensity factor, ΔK vs. number of loading cycles. The three near-threshold crack propagation regimes are shaded in yellow.

Figure 10 shows crack length, resistance change in the sensor, and stress intensity range calculated from the applied loads vs. number of applied load cycles. The near-threshold regimes are shaded in yellow and correspond directly to the shaded regions in Figure 5b. In the near-threshold regimes, CA cyclic loads were designed to keep the range of stress-intensity factors, ∆K, close to the near-threshold stress intensity factor of A36 structural steel, which was found to be approximately 3 MPa-m^1/2^ [57]. In the near-threshold crack growth regimes, the fatigue crack did not grow notably (black line (thinner) in Figure 10), which is consistent with the 3D-DIC measurements (discussed regarding Figure 9). The resistance change in the CNT sensor (solid red line in Figure 10) follows the same trend, also with no significant resistance change occurring during the near-threshold crack growth regimes. Thus, fatigue crack activity can be monitored using the resistance response of the sensor and the CNT fatigue crack sensor does not report false positives under near-threshold crack propagation conditions.

### 3.3. Re-Initiation at a Crack-Stop Hole

**Figure 11:**
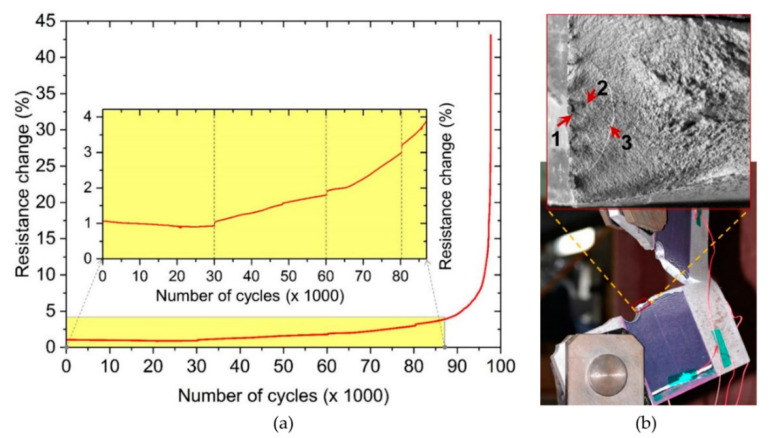
Crack re-initiation from a crack-stop hole: (**a**) resistance change (%) vs. number of applied load cycles. Dashed lines in inset show the number of load cycles corresponding to beach marks; (**b**) failure of the specimen with crack-stop hole beach marks (inset) marked by arrows numbered 1 to 3.

The objective of this test was to evaluate the ability of the CNT sensor to capture crack re-initiation at a crack-stop hole. Crack-stop holes are drilled near the tip of a fatigue crack to reduce the stress intensity and remove material that may have strain-hardened. The idea is that this arrests the crack and thus increases the remaining fatigue life of the structure [8]. Figure 11a shows the measured resistance change of the CNT-based sensor throughout this test. From load cycles 1 to 30 k, the resistance remains approximately constant (at about 1%, see inset in Figure 11a). The fracture surface of the specimen indicates that the first beach mark was created after 30 k cycles (arrow #1 in Figure 11b), which implies that this is when the fatigue crack re-initiated. Subsequent beach marks were also visible on the surface, and it was observed that the crack grew nearly through the thickness of the substrate after 80 k cycles (arrow #3). The sensor was able to capture crack nucleation by showing an increase in resistance change after 30 k cycles (see inset in Figure 11a). This demonstrates that the CNT fatigue sensor can successfully capture re-initiation of a fatigue crack from a crack-stop hole as well as propagation following re-initiation of the hole even before the crack has reached the outer surface. Once the fatigue crack reaches the surface, the resistance dramatically increases, which was around 95 k load cycles.

## 4. Conclusions

This article documents the effectiveness of a CNT-based sensor that is flexible and can thus be utilized for a wide range of fatigue sensitive details found in real structures. The cycle-by-cycle relationship between the response of the CNT-based fatigue sensor and crack length was used in the sensor evaluation processes. The loading protocols employed in the experimental tests were able to simulate real-life crack growth scenarios that include near-threshold crack propagation and crack re-initiation scenarios, as well as continuous crack propagation at various rates. Crack length quantification based on BFS measurements was used to verify the measurements from the proposed sensor. Additionally, these calculated crack lengths were verified with those found from forensic investigations of the fatigue-fractured surface. It was demonstrated that the CNT fatigue crack sensor allows monitoring of metal fatigue cracks continuously and in real time as well as being robust, i.e., not giving false positive readings, under near-threshold crack propagation conditions, i.e., where the crack is quasi-stationary. Furthermore, the proposed sensor is able to detect re-initiation of a crack even before it has reached the surface of the member. In the near-threshold test the sensitivity of the resistance change to crack growth is higher than the sensor used in the continuous crack detection test, which correlates with the difference in initial sensor resistance. Finally, the sensor can continuously monitor a growing fatigue crack of virtually any length, provided adequate length of the sensor.

The sensing principle is based on measuring the electrical resistance of the sensor, which is simple to implement, offering real-time monitoring capabilities. Thus, development of a field-deployable data acquisition system is straight forward and can be done using inexpensive open-source components. Future work includes evaluating the sensor on a real structure under actual temperature and humidity conditions over the long term using such a data acquisition system.
